# Supplementation with Sunflower/Fish Oil-Containing Concentrates in a Grass-Based Beef Production System: Influence on Fatty Acid Composition, Gene Expression, Lipid and Colour Stability and Sensory Characteristics of Longissimus Muscle

**DOI:** 10.3390/foods11244061

**Published:** 2022-12-15

**Authors:** Aidan P. Moloney, Shane McGettrick, Peter G. Dunne, Kevin J. Shingfield, Robert Ian Richardson, Frank J. Monahan, Finbar J. Mulligan, Marion Ryan, Torres Sweeney

**Affiliations:** 1 Teagasc, Animal & Grassland Research and Innovation Centre, Grange, Dunsany, Co., C15PW93 Meath, Ireland; 2 School of Agriculture and Food Science, University College Dublin, D04V1W8 Dublin, Ireland; 3 School of Veterinary Medicine, University College Dublin, D04V1W8 Dublin, Ireland; 4 Teagasc Food Research Centre, Ashtown, D15KN3K Dublin, Ireland; 5 Natural Resources Institute Finland, 00790 Helsinki, Finland; 6 Division of Farm Animal Science, School of Clinical Veterinary Science, University of Bristol, Langford, Bristol BS40 5DU, UK

**Keywords:** beef, muscle, CLA, omega-3 fatty acids, shelf-life

## Abstract

Beef contains an array of conjugated linoleic acid (CLA) isomers for which positive effects have been reported in animal models of human disease. The objectives were to develop a CLA-enriched beef production system and to assess its quality. Sixty Spring-born heifers were housed in Autumn and offered unwilted grass silage and a barley/soyabean concentrate or wilted grass silage and a concentrate containing sunflower oil and fish oil. In May, both groups were offered either pasture for 22 weeks, restricted pasture and sunflower oil and fish oil for 22 weeks, or pasture for 11 weeks and restricted pasture and sunflower oil and fish oil for the final 11 weeks. The predominant CLA isomer in beef was cis9, trans11 representing on average, 80% total CLA. The modified winter diet followed by supplementation for 22 weeks resulted in beef that had a CLA concentration that was higher, at a comparable intramuscular fatty acid concentration, than previously reported. The lipid and colour stability (over 10 days in modified atmosphere packaging) and sensory characteristics were generally not negatively affected. There were minor effects on the expression of candidate genes involved in lipid metabolism. Consumption of this beef would make a substantial contribution to the quantity of CLA suggested to have a positive effect on consumer health.

## 1. Introduction

The interest of consumers in the relationships between diet and well-being has resulted in a growing preference for foods which are perceived as offering health benefits [1]. While beef is perceived to have a high proportion of saturated fatty acids (SFA), it also contains polyunsaturated fatty acids (PUFA), particularly omega-3 PUFA, which are beneficial to human health [2]. Beef also contains an array of conjugated linoleic acid (CLA) isomers, products of ruminal biohydrogenation of dietary PUFA [2] of which the cis9, trans11 isomer is most prominent. Positive effects of CLA are widely reported in animal models of human disease and supported, albeit not to the same extent, by human studies [3,4]. A desire to enhance the nutritional value has focused on strategies to increase the concentration of CLA in beef. The most effective strategy has been to modify the diet of the animal by changing the basal ration and/or supplementing with PUFA, in particular sources of linoleic acid such as sunflower oil or safflower oil [5]. In an indoor context, wilted rather than unwilted grass silage [6] and supplementation with sunflower oil alone (Ref. [7] or with fish oil [8] enhanced the CLA concentration in beef muscle. Compared to concentrates, consumption of grazed grass generally results in an increase in the deposition of CLA in muscle [2] and supplementing grazed grass with sunflower oil alone [8] or with a blend of sunflower oil and fish oil [9] increased the concentration still further. Most CLA enrichment studies, however, have been carried out close to the slaughter of cattle and not in a production system context. The first objective of this study was to integrate the above findings within one grass-based beef production system, in which weaned Spring-born suckled heifers spend the first winter indoors and are subsequently slaughtered from pasture at 20 months of age.

Due to the presence of unsaturated fatty acids, muscle foods are inherently susceptible to lipid oxidation and this susceptibility increases as the number of double bonds increases [10]. Changes in both the concentration and composition of fatty acids may therefore affect the colour and lipid stability of beef during retail display and also its sensory characteristics [11]. Deleterious effects on shelf life or sensory characteristics would diminish the benefits of nutritional enhancement of beef. Accordingly, the second objective was to determine the effect of CLA enhancement on these beef quality variables.

Since PUFA supplementation can influence the expression of genes coding for enzymes involved in lipid metabolism [12], the third objective was to determine the effect of the modifications to the heifer beef production system, on the expression of selected lipid-related genes in muscle and their relationship with the lipids themselves. This might suggest more targeted future strategies for CLA enrichment of beef.

## 2. Materials and Methods

### 2.1. Silage Preparation

Both silages were prepared from the same perennial ryegrass-based sward. Alternative strips of cut grass were either ensiled directly and treated with 3l Addsafer (Interchem Ltd., Dublin, Ireland) (48% formic acid and 16% ammonium tetraformate/l)/tonne of grass (unwilted) or wilted for 32 h, turned once and then ensiled without an additive. During the wilting period, there was no rainfall. Maximum and minimum temperatures on the day of cutting were 26.0 and 12.1 °C, respectively. The corresponding temperatures during the day of ensiling of the wilted grass were 26.3 and 13.6 °C.

### 2.2. Animals and Management

This study was carried out under licence from the Irish Government Department of Health and Children and with the approval of Teagasc, the Agricultural and Food Development Authority. All procedures complied with national regulations concerning experimentation on farm animals.

Sixty Spring-born Charolais-sired crossbred heifers were purchased in October from Irish farms and brought to Teagasc, Animal and Grassland Research and Innovation Centre, Grange, Dunsany, County Meath, Ireland. They were offered unwilted grass silage ad libitum and 1 kg proprietary concentrate which contained per kg, 865 g rolled barley, 65 g soyabean meal, 45 g cane molasses and 25 g mineral + vitamin mix, until the start of the experiment. The animals were weighed and blocked (10) according to body weight (BW) (mean initial BW 300 kg, SD = 23.0 kg) on December 14. Within block, the heifers were assigned at random to receive either a control or CLA promoting diet. The control diet consisted of unwilted grass silage offered ad libitum and 1.5 kg of the above “standard” concentrate (US). As the experiment progressed, the concentrate allowance was increased, based on periodic weighing of the cattle, to achieve an average daily gain (ADG) target of 0.6 kg/d. The CLA diet consisted of a restricted amount of the wilted silage and 1.5 kg of a concentrate that contained per kg, 137 g sunflower oil, 58 g fish oil (derived from a mix of mackerel and herring oil), 49 g cane molasses,46 g soyabean meal, 686 g pollard and 24 g mineral + vitamin (20,000 IU vitamin E/kg) mixture (WO). As the winter phase of the experiment progressed, the allowance of the CLA concentrate was increased to ensure similar growth rate in both groups while maintaining the oil allowance at a maximum of 50 g/kg dietary dry matter (DM). The animals were also pre-assigned to post-winter treatments of (1) unsupplemented grazed grass from turnout for 22 weeks (G0), (2) unsupplemented grazed grass for 11 weeks and then a CLA supplement for 11 weeks (G11), or (3) grazed grass and a CLA supplement for 22 weeks (G22). 

During the winter, the heifers were penned in groups of 3 or 4 according to winter diet (9 pens/diet) balanced for block as far as was practicable. Fresh feed was offered daily and the weight of any uneaten feed recorded and removed. 

From early-May, the G0 and G11 animals rotationally grazed a predominantly *Lolium perenne* L. pasture as 4 groups of 10 animals/group (two groups from each winter diet). The daily DM allowance of 25 g kg^−1^ BW was achieved by measuring pre-grazing grass mass using a rising plate meter (Filips Folding Plate Meter, Jenquip, New Zealand) and calculating the amount of grass available. The area required to supply the grass allowance (without access to the previous grazing area) was then calculated. Animals were offered a fresh allowance every 2 to 3 days or more frequently depending on rainfall. The two groups of G22 animals separately grazed a smaller area to compensate for the high energy concentrates, and were offered a daily concentrate allowance of 2.5/425 kg bodyweight. The concentrate initially contained per kg, 133 g sunflower oil, 67 g fish oil (derived from a mix of mackerel and herring oil), 50 g cane molasses, 725 g pollard and 25 g mineral + vitamin (20,000 IU vitamin E/kg) mixture. Due to slow consumption, after 1 month 200 g of pollard was replaced by rolled barley and by a further 100 g 10 days later. The grass allowance was adjusted according to growth of the animals to ensure a similar rate of carcass growth. The concentrates were offered individually using an auto-locking feeding trailer and uneaten feed removed and weighed. Samples of grass and concentrates were taken daily and twice weekly, respectively. All samples were stored at −20 °C for chemical and fatty acid analysis.

### 2.3. Slaughter and Sample Collection

Animals were slaughtered at Meadow Meats Ltd., Rathdowney, County Laois, Ireland. The animals were weighed the day before slaughter and slaughtering commenced 45 min after arrival at the abattoir. All slaughter and dressing procedures complied with Regulations (EC) No. 1099/2009 and No. 853/2004 and electrical stimulation was not applied. Post slaughter, carcasses were weighed and graded for conformation and fatness. Approximately 30 min post-mortem, a sample (50 g approx.) of longissimus muscle (LM) and subcutaneous adipose tissue was removed from each carcass from above the 11th and 12th rib for subsequent gene expression analysis. The samples were dissected aseptically into smaller pieces and stored in RNALater™, (Ambion Ltd., Cambridge, UK) for 24 h. Subsequently, the RNALater™ was removed and the samples were stored at −80 °C. Carcasses were then placed in a chill.

At 24 h post-mortem, the right LM from the 10th rib to the posterior end (3 rib striploin commercial cut,) was excised from each carcass. The muscle was vacuum packaged (Webomatic^®^ vacuum-packaging systems Super Vax, ThyssenKrupp Schulte GmbH, Düsseldorf, Germany) and transferred to Teagasc Food Research Centre, Ashtown, Dublin and stored at 2 °C. At 48 h post-mortem, pH was measured at the 10th rib area by making a scalpel incision approximately 2 cm into the LM and inserting a pH electrode (EC-2010-11, Reflex Sensors, Ltd., Dublin, Ireland) connected to a portable pH meter (Model No. 210A, Thermo Electron Corp., Orion Products, Beverly MA, USA) set to record at 5 °C. The pH electrode was calibrated using buffers of pH 7.00 and 4.00 and rinsed between measurements. A section of LM (15 cm) was then vacuum packaged, aged for 14 days (2 °C) and stored at −20 °C pending sensory assessment. A further section (20 cm) was removed, vacuum packaged and aged for a further 19 days (21 days aging in total) for colour stability measurement. Finally, two steaks (25 mm thickness) were removed, vacuum packaged and stored at −20 °C pending chemical and fatty acid analysis, respectively.

### 2.4. Colour Stability Measurement

Six steaks of 2.5 cm were dispensed into styrofoam trays (187 × 145 × 40 mm, Lin Fresh™, Linpac Plastics, Ltd., Knottingley, West Yorkshire WF11 0BL, UK) to which absorbent pads had been added. Trays were sealed under oxygen impermeable barrier film (Versatile Packaging, Ltd., Castleblayney, Co., Monaghan, Ireland) (oxygen transmission rate: 8 cm^3^ O_2_/m^2^/24 h at 23 °C and 75% relative humidity) following evacuation and flushing with 80%O_2_:20%CO_2_ (Food Fresh™, BOC Gases, Dublin, Ireland). A modified atmosphere packaging (MAP) machine (Foodpack 400 V/G, Ilpra^®^ S.p.A., Corso Pavia 30, Vigevano 27029, Pavia, Lombardia, Italy) was used. Prior to packaging, the gaseous composition of inflated empty trays was checked using an automated MAP testing device (MAPtest 3050 Packaging atmosphere analyser, Hitech Instruments Ltd., Luton, Hertfordshire LU4 8EF, UK). Trays were randomly positioned in an open-fronted retail display cabinet with a regular automatic defrost cycle (Cronos fan-assisted cabinet, Crisobanc Refrigeration, Crisobanc S.p.A., 35038 Torreglia, Padova, Italy) under permanent fluorescent lighting (Philips™ TL-D 58W, Philips Electronics (Ireland) Ltd., Fonthill Rd., Dublin 22, Ireland; luminous flux of 2800 lm, lux = 3616). The trays were shielded using an insulating blind, to maintain a uniform temperature distribution throughout. Hunter colour coordinates (‘L’ (lightness), ‘a’ (redness), ‘b’ (yellowness)) were measured on day 0 (approximately 3 h after packaging) and on days 1, 3, 6, 8 and 10 using a benchtop Hunter lab UltraScan XE spectrocolorimeter with Universal Software Version 2.2.2 (Hunter Laboratories, Reston, VA, USA). The ‘C’ (saturation) and ‘H’ (hue angle) values were calculated as (a^2^ + b^2^)^0.5^ and [tan^−1^(b/a)] [180/π], respectively. Reflectance spectra from 400 to 700 nm at 10 nm intervals were recorded. These data were used to estimate pigment proportions [13] and the difference between reflectance at 630 nm and 580 nm (R_630_–R_580_) as indices of discolouration. Lipid oxidation on days 0 and 10 was assessed as 2-thiobarbituric acid reactive substances (TBARS). 

### 2.5. Chemical Analysis

Fat was extracted from 2 g homogenised LM, separated into the neutral lipid (NL) and polar lipid (PL) fractions and fatty acids methylated as previously described [14]. Fatty acid methyl esters (FAME) were analysed using a Varian 3500 GLC (Varian, Harbor City, CA, USA) and a 100 m CP-Sil 88 column (100 m × 0.25 mm i.d., 0.2 µm film thickness, Supelco, Bellefonte, PA, USA). Hydrogen was the carrier gas and GC conditions were as described in [15]. Individual FAME were identified by retention time with reference to external standards (Supelco 37 component FAME Mix, Supelco Inc., Bellefonte, PA, USA). Individual standards from Matreya (Matreya Inc., Pleasant Gap, PA, USA) were used for identification of FAME not contained in the mix. Fatty acids for which no commercial standards were available had been identified in identical chromatographic conditions as in the present study, by Shingfield et al. [16] using 4,4-dimethyloxazoline derivatives and analysed by GC-MS. The appropriate retention times were used to identify these fatty acids in the present analysis. Individual FAME were quantified by using C23:0 as the internal standard. 

For CLA methyl ester analysis, FAME were evaporated under nitrogen, dissolved in heptane and analysed by HPLC using four silver-impregnated silica columns (ChromSpher 5 lipids, 250 × 4.6 mm; 5 μm particle size, Varian Ltd., Walton-on-Thames, UK) coupled in series and 0.1% (*v*/*v*) acetonitrile in heptane as the mobile phase [17]. Isomers were identified using an authentic CLA methyl ester standard (O-5632, Sigma-Aldrich St. Louis, MO, USA) and chemically synthesised trans-9, cis11 CLA [17]. Identification was verified by cross-referencing with the elution order reported in the literature [18] using cis9, trans11 CLA as a landmark isomer. Vitamin E and TBARS concentrations were measured as described in [19].

The general composition of feeds was determined as previously described [20]. The fatty acid composition of feeds was determined using the procedure described in [21] with the minor modification that toluene was used instead of benzene. 

### 2.6. RNA Purification, cDNA Synthesis and Quantitative Polymerase Chain Reactions (QPCR)

RNA was extracted and purified from 100 mg of tissue in Tri-Reagent (Sigma-Aldrich, St. Louis, MO, USA), followed by a DNase step (Promega, Madison, WI, USA). The total RNA was quantified and assessed for purity on a NanoDrop Spectrophotometer ND1000 (Thermo Scientific, Wilmington, DE, USA). All cDNA synthesis was carried out using 1 μg of total RNA using Superscript™ III First-Strand Synthesis kit for RT-PCR (Invitrogen Life Technologies, Carlsbad, CA, USA) and random hexamers according to the manufacturers’ instructions. 

Primer sets for peroxisome proliferator activated receptor alpha (PPARα), Δ9 stearoyl-CoA desaturase (SCD), sterol regulatory element binding protein 1 (SREBP1), SREBP chaperone (SCAP), peroxisome proliferator activated receptor gamma (PPARγ), fatty acid synthase (FAS), lipoprotein lipase (LPL), Δ6 desaturase/fatty acid desaturase 2 (FADS2), ribosomal protein (RPL0) and glyceraldehyde-3-phosphate dehydrogenase (GAPDH) genes were designed using Primer express^TM^ (Applied Biosystems, Foster City, CA, USA) (Supplementary Table S1). Primer sets were synthesised by Eurofins (Milton Keynes, UK). Specificity of the primers was established in silico using BLAST and confirmed by examining the dissociation curves for each primer set. The QPCR assay efficiencies were determined by plotting the cycling threshold (CT) values resulting from 4-fold serial dilutions of cDNA against their arbitrary quantities and only assays demonstrating 90–110% efficiency and single products were accepted in this analysis. QPCR assays were performed on cDNA in 96-well optical plates on 7500 ABI Prism Sequence Detection System (PE Applied Biosystems, Foster City, CA, USA). For each 20 µL reaction, 5 µL cDNA, 1.2 µL (forward and reverse primer, 5 µM), 10 µL SYBR Green PCR Master Mix (PE Applied Biosystems, Foster City, CA, USA) and 3.8 µL molecular grade water were used. The two step PCR program was as follows: 95 °C for 10 min, 1 cycle, then 60 °C for 1 min, 95 °C for 15 s, 40 cycles. 

Normalised relative quantities were obtained using the software, qbase PLUS (Biogazelle, Ghent, Belgium) from stable reference genes; GAPDH and RPL0. These genes were confirmed to have M values (<1.5) as calculated by the GeNorm algorithm within qbase PLUS. 

### 2.7. Sensory Assessment 

Samples were arranged according to the blocking structure of the experimental design. The day before sensory assessment, loin sections were thawed in a refrigerator set at 1 °C. On the morning of sensory assessment, loin sections were removed from their packs and steaks 1.9 cm thick were cut. Steaks were cooked under a conventional grill, turning every three minutes, until the internal temperature of the muscle reached 74 °C as measured by a thermocouple probe (Testo Limited, Alton, UK). Ten samples, (2 cm × 2 cm × 1.9 cm) were then cut from the approximate centre of the steaks avoiding incursions of connective tissue where present, wrapped in pre-labelled foils, placed in a heated incubator at 65 °C and served hot to a 10-person trained professional taste panel, using the same people for the duration of each experiment. Assessors scored individual flavours using 0–100 mm unstructured intensity line scales where 0 = nil and 100 = extreme. Assessments took place in a purpose-built panel room illuminated by red light. Each booth contained a computer screen and optical mouse as part of the computerised sensory system (Fizz, Version 2.20 h, Biosystemes, Couternon, France), for direct entry of sensory responses. At each session, assessors tasted 6 samples of loin steaks in balanced order such that first order carry over effects were reduced [22]. 

### 2.8. Data Calculations and Statistical Analysis

Daily grass DM intake was estimated based on the growth of the animals and their associated energy requirement [23]. The fatty acid concentrations in total muscle were calculated as the sum of the fatty acid concentrations in the NL and PL fractions. Selected nutritionally relevant fatty acid indexes were calculated, according to Ulbricht and Southgate [24]. Data were analysed according to a split plot design using Genstat (19th edition, VSN International, Hemel Hempstead, UK). The model had block and winter ration in the main plot and summer ration and all winter by summer interactions in the subplot. The effect of duration of concentrate supplementation was examined using orthogonal polynomials. For colour data relating to retail display, the design was a split-split plot. The split plot was as described above with time of display and all time-related interactions in the sub subplot. Gene expression data that were not normally distributed were transformed using the appropriate lambda function determined through the Box Cox transformation (ABOXCOX procedure in Genstat) and analysed as a split-plot design as described above. Multiple analysis of variance using SAS was used to calculate partial correlation coefficients (*p*), from the error sum of squares and cross products (SSCP) matrix, between selected concentrations of fatty acids and transformed gene expression data in muscle. 

## 3. Results

### 3.1. Chemical Composition (Table 1)

The wilted silage tended to have a higher DM and a lower oil concentration but the chemical composition was generally similar to that of the unwilted silage. The grass tended to have a higher digestibility and tended to have the highest proportion of C18:3 compared to the silages. The oil-rich concentrates averaged 233 g oil/kg DM which had a higher proportion of C18:2 than the standard concentrate. The inclusion of fish oil was reflected in the proportions of C20:5 and C22:6 detected in the oil-rich concentrates compared to the standard concentrate. 

**Table 1:** Chemical composition (mean (standard deviation)) and estimated metabolisable energy concentration of the forages and concentrates.

	Standard Concentrate	Winter Oil	Unwilted Silage	Wilted Silage	Grass	Summer Oil
Dry matter (DM, g/kg)	830 (9.1)	884 (10.7)	184 (65.0)	461 (63.6)	198 (44.8)	892 (9.7)
Ash ^1^	50 (17.9)	65 (8.6)	116 (15.4)	111 (8.9)	88 (14.6)	64 (3.6)
Crude protein ^1^	148 (16.8)	161 (8.2)	156 (14.4)	160 (12.1)	178 (42.4)	124 (4.1)
Oil B ^1,2^	29 (1.3)	235 (18.8)	41 (4.8)	29 (4.2)	26 (8.1)	231 (5.1)
NCGD ^1,3^	936 (37.1)	844 (21.6)	-	-	-	883 (4.5)
ME (MJ/kg DM) ^4^	11.6 (0.55)	16.4 (0.46)	10.2 (0.59)	10.3 (0.40)	11.5 (0.07)	16.8 (0.18)
DM digestibility (g/kg)	-	-	700 (37.6)	707 (25.7)	781 (53.2)	-
pH	-	-	4.4 (0.18)	4.4 (0.30)	-	-
Ammonia ^1^	-	-	2.1 (0.41)	0.7 (0.22)	-	-
Lactic acid ^1^	-	-	24 (15.0)	37 (13.4)	-	-
Fatty acids (g/kg fatty acids)						
C14:0	1.8 (0.39)	10.8 (2.80)	6.1 (1.10)	5.7 (0.70)	7.0 (1.40)	12.9 (2.10)
C14:1	0.2 (0.81)	5.1 (3.50)	1.8 (2.4)	0.3 (0.51)	-	2.2 (3.22)
C16:0	298.0 (34.50)	86.7 (9.10)	143.1 (12.09)	145.3 (4.29)	147.2 (12.16)	94.0 (6.81)
C16:1	-	9.8 (3.21)	5.3 (3.11)	2.6 (0.24)	1.2 (6.11)	11.8 (2.73)
C18:0	31.6 (9.10)	29.9 (1.04)	19.2 (4.64)	19.9 (2.56)	21.7 (4.90)	30.8 (1.22)
C18:1	150.6 (12.70)	201.1 (5.72)	32.1 (21.01)	22.5 (12.42)	26.1 (7.20)	211.7 (11.33)
C18:2	417.7 (60.41)	527.7 (35.41)	144.1 (41.38)	123.5 (9.12)	110.1 (1.43)	481.5 (41.24)
C18:3	33.4 (8.70)	9.5 (1.02)	373.3 (33.65)	394.4 (29.72)	404.4 (62.41)	16.2 (18.61)
C20:0	1.4 (2.00)	0.7 (1.52)	4.7 (1.11)	4.5 (0.51)	3.7 (1.06)	1.5 (2.11)
C20:1	0.7 (2.22)	19.7 (5.62)	3.2 (2.97)	1.0 (0.57)	0.6 (0.82)	24.3 (3.62)
C20:5	0.4 (1.11)	12.5 (3.52)	0.9 (1.44)	0.5 (0.86)	-	14.9 (3.44)
C22:6	-	19.7 (5.82)	0.7 (1.92)	0.1 (0.27)	0.2 (0.52)	24.0 (7.22)

^1^ g/kg DM. ^2^ Acid hydrolysis, ether extract. ^3^ Neutral cellulase gammanase digestibility. ^4^ Metabolisable energy; for concentrates = 0.014 × NCGD + 0.025 Oil b [25], for silage and grass = 0.16 × [(0.98 × DM Digestibility (%)) − 4.8] [26].

### 3.2. Animal Performance, Longissimus Muscle Colour and Chemical Composition (Table 2)

All stated differences in this and subsequent sections were statistically significant (*p* < 0.05).

The mean liveweight of the animals offered the US and WO rations at the end of the 140-day winter phase was 385 and 387 (sed = 5.5) kg, respectively. The corresponding growth rates were 611 and 608 (sed = 35.5) g/day. During this phase, the animals offered the US and WO rations consumed (on a pen basis) 3.42 kg of unwilted silage and 4.35 kg wilted silage DM (sed = 0.063), respectively, per head daily. The corresponding consumption of concentrates was 1.52 kg and 1.41 kg DM (sed = 0.029). During the relevant grazing phases, the daily concentrate intakes for USG11, USG22, WOG11 and WOG22 were 3.6, 3.9, 3.3 and 3.7 g per kg BW (sed = 0.216), respectively. The estimated daily grass DM intakes were 7.9 and 5.1 kg for USG0 and USG22 and 7.8 and 5.3 kg for WOG0 and WOG22, respectively. For USG11 and WOG11, estimated daily grass DM intakes during the first 11 weeks after turnout were 9.5 and 9.0 kg, respectively and 4.4 and 4.5 kg, respectively during the supplementation phase. Growth rate from turnout to slaughter, carcass weight and carcass fat classification increased linearly with duration of concentrate supplementation. There was no difference between treatments for LM pH and colour and for LM moisture, lipid or vitamin E concentrations. The LM from 4 carcasses had high pH (<6.0) with associated lower L value (L < 33.3). When these samples were excluded from the analysis, pH was unchanged but redness (a value) and chroma increased linearly with duration of concentrate supplementation.

**Table 2:** Growth, carcass and muscle characteristics of beef heifers.

Winter Ration (W)	Control			Oil-Enriched				Significance ^1^		
Duration of Supplementation (Weeks D)	0	11	22	0	11	22	sed	W	D ^1^	Wx × D
Weight (kg)										
End of winter (turnout)	379	378	397	382	392	385	8.8	NS	NS	NS
After11weeks	462	473	481	457	469	472	7.8	NS	L *	NS
Pre-slaughter	492	512	524	491	507	515	13.0	NS	L **	NS
Growth (g/kg)										
Turnout–11 weeks	918	1063	933	833	888	938	75.8	*	NS	NS
11 weeks–slaughter	387	496	560	459	445	583	64.1	NS	L *	NS
Turnout-slaughter	670	798	762	647	684	773	62.7	NS	L *	NS
Carcass										
Weight	249	267	278	246	269	270	7.40	NS	L ***	NS
Fatclass ^2^	2.70	2.85	3.05	2.50	3.00	3.28	0.319	NS	L **	NS
Conformation class ^3^										
Longissimus muscle										
pH	5.57	5.61	5.76	5.56	5.60	5.57	0.113	NS	NS	NS
pH ^4^	5.53	5.53	5.54	5.56	5.60	5.57	0.065	NS	NS	NS
Colour ^5^										
L	37.1	38.0	36.8	38.2	38.0	38.7	1.22	NS	NS	NS
a	16.9	17.5	17.1	17.2	17.3	18.7	1.09	NS	NS	NS
b	9.0	9.5	9.0	9.4	9.3	10.0	0.62	NS	NS	NS
C	19.1	20.0	19.3	19.6	19.7	21.2	1.24	NS	NS	NS
H	28.1	28.6	27.7	28.7	28.3	28.2	0.60	NS	NS	NS
Colour ^4,5^										
L	37.7	39.0	38.0	38.2	38.0	38.7	0.89	NS	NS	NS
a	17.6	18.4	18.5	17.2	17.3	18.7	0.77	NS	L *	NS
b	9.3	10.1	9.7	9.4	9.3	10.0	0.44	NS	NS	NS
C	19.9	21.0	20.9	19.6	19.7	21.2	0.85	NS	L *	NS
H	28.0	28.7	27.6	28.7	28.3	28.2	0.62	NS	NS	NS
Longissimus muscle composition (g/kg)										
Moisture	725	728	723	729	728	723	4.7	NS	NS	NS
Lipid	34	34	35	28	29	31	61	NS	NS	NS
Vitamin E	3.6	3.4	3.4	3.4	3.6	4.0	0.33	NS	NS	NS

^1^ sed = standard error of the difference for the Wx × D interaction with n = 10/group; L, Q are linear and quadratic effects of duration of supplementation, respectively, * = *p* < 0.05, ** = *p* < 0.01, *** = *p* < 0.001. ^2^ Where 1 = very lean, 5 = very fat. ^3^ Where 1 = poor, 5 = very well conformed. ^4^ Four samples with pH > 6.0 removed. ^5^ L = lightness, a = redness, b = yellowness, C = chrome, H = hue.

### 3.3. Muscle Fatty Acid Composition

#### 3.3.1. Fatty Acid Concentrations (Table 3)

Neither the total fatty acid concentration, the concentration of any of the main fatty acids listed in Table 3 (>1 mg/100 g muscle for all treatment means) or the nutritional indices were affected by the winter ration. There tended (*p* = 0.056) to be a quadratic response to duration of concentrate supplementation for the total fatty acid concentration such that the concentration was lower after 11 weeks of concentrate supplementation (3009 mg/100 g tissue) compared to none (3346 mg/100 g tissue) or 22 weeks (3883 mg/100 g tissue). Increasing the duration of concentrate supplementation linearly increased the concentrations of C16:0 (quadratic), C16:1trans11, C17:0iso + C16:1trans9, C18:1trans11, C18:1cis16, C18:1trans10, C18:1trans12, C18:1trans9, C18:9,14, C18:2 10,13, C18:2cis9, cis12 (linoleic acid, LA), C18:2trans11,cis15, C18:3cis9,trans11,cis15, C20:0, C20:1, C20:4cis8,cis11,cis14,cis17, C22:0, C22:1cis11,C22:6cis4,cis7,cis10,cis13,cis16,cis19 (docosahexaenoic acid, DHA, also quadratic),CLA cis9, trans11, total CLA isomers, PUFA and omega-6 PUFA. 

Increasing the duration of concentrate supplementation linearly increased the PUFA: SFA (also quadratic) and omega-6: omega-3 PUFA ratios and linearly decreased the concentrations of C16:1trans12 (quadratic only) and CLA trans10,cis12 and the thrombogenic index (also quadratic). 

There was an interaction (linear) between the winter ration and the duration of concentrate supplementation such that the concentration of C16:1cis9 and C18:1trans13 decreased with the increase in duration of concentrate supplementation for the US diet but increased for the WO diet. For the concentration of C18:3cis9,cis12,cis15 (linolenic acid LNA), while the mean value was higher for the US compared to the WO winter ration and linearly decreased with the increase in the duration of concentrate supplementation, the decrease was greater for US ration.

**Table 3:** The concentration of fatty acids (mg/100 g muscle) in *longissimus* muscle of beef heifers.

Winter Ration (W)	Control			Oil-Enriched				Significance ^1^		
Duration of Supplementation (Weeks D)	0	11	22	0	11	22	sed	W	D	W × D
C14:0	73	47	66	44	45	75	19.2	NS	NS	NS
C14:1	24	13	18	13	12	19	5.9	NS	NS	NS
C15:0	18	12	16	12	13	18	3.6	NS	NS	NS
C15:0anteiso	8	5	7	5	5	8	1.6	NS	NS	NS
C15:1	7	5	6	5	5	6	1.5	NS	NS	NS
C16:1trans12	7	4	5	5	4	5	1.1	NS	Q *	NS
C16:0	802	536	707	550	528	749	156.1	NS	Q *	NS
C16:0iso	7	4	5	4	4	5	1.2	NS	NS	NS
C16:1cis9 ^2^	119	67	88	66	64	100	25.7	NS	Q *	L *
C16:1trans7 + trans8	13	10	14	11	11	14	2.7	NS	NS	NS
C16:2cis9,cis12	6	4	3	4	4	4	0.9	NS	L *	L *
C16:1cis13	4	2	3	2	2	3	1.0	NS	NS	NS
C16:1t11	2	2	3	2	2	3	0.6	NS	L **	NS
C17:0	35	23	29	23	23	30	6.4	NS	Q+	NS
C17:0iso + C16:1trans9	5	8	9	6	9	11	1.3	NS	L ***	NS
C17:1cis9	18	13	16	12	13	18	4.1	NS	NS	NS
C18:0	469	326	432	362	354	481	85.9	NS	Q+	NS
C18:0ante	1	1	1	1	1	2	0.2	NS	NS	NS
C18:1trans11	80	140	258	115	160	285	51.2	NS	L ***	NS
C18:1cis15 ^3^	5	4	6	3	5	6	1.3	NS	L+	NS
C18:1cis16	10	8	11	7	8	13	2.7	NS	L *	NS
C18:1cis11	29	12	25	11	13	15	8.5	NS	Q+	NS
C18:1cis12	9	3	5	2	4	4	4.1	NS	NS	NS
C18:1cis13	8	4	7	4	4	6	2.1	NS	NS	NS
C18:1cis9	1148	732	925	785	741	1030	220.2	NS	Q *	NS
C18:1trans10	8	11	18	9	16	22	5.1	NS	L **	NS
C18:1trans12	7	11	20	10	18	25	5.1	NS	L ***	NS
C18:1trans13	66	31	42	26	44	46	15.5	NS	NS	L *
C18:1trans16	8	7	10	6	7	9	1.9	NS	L+	NS
C18:1trans9	4	5	9	5	7	12	2.1	NS	L ***	NS
C18:29,14	3	2	3	2	3	4	0.7	NS	L *	NS
C18:2,10,13 ^4^	9	7	10	6	8	12	2.5	NS	L *	NS
C18:2cis9,cis12	61	63	76	52	60	78	6.6	NS	L ***	NS
C18:2trans11,cis15	12	12	17	10	12	17	3.4	NS	L *	NS
C18:3cis9,cis12,cis15	33	23	21	25	22	22	3.1	*	L ***	L *
C18:3cis9, trans11,cis15	1	2	2	1	2	2	0.4	NS	L **	NS
C18:2:trans12,cis15	3	2	2	2	2	3	0.8	NS	NS	NS
C20:0	4	4	7	4	5	9	1.4	NS	L ***	NS
C20:1	5	8	13	5	7	13	2.7	NS	L ***	NS
C20:2 c11,c14	2	2	3	2	2	4	0.5	NS	L **	*
C20:3c11,c14,c17	1	1	1	1	1	1	0.3	NS	L *	NS
C20:3cis8,cis11,cis14	4	4	3	3	4	4	0.5	NS	NS	NS
C20:4cis5,cis8,cis11,cis14	12	12	10	10	12	10	1.5	NS	Q+	NS
C20:4cis8,cis11,cis14,cis17	2	3	3	2	3	3	0.5	NS	L **	NS
C20:5cis5,cis8,cis11,cis14,cis17	8	9	8	9	9	9	1.2	NS	NS	NS
C22:0	1	1	2	1	1	2	0.4	NS	L **	NS
C22:1cis11	1	3	7	2	3	7	1.5	NS	L ***	NS
C22:5cis7,cis10,cis13,cis16,cis19	12	11	10	11	11	10	1.2	NS	L+	NS
C22:6cis4,cis7,cis10,cis13,cis16,cis19	1	2	2	1	2	3	0.5	NS	L *** Q *	NS
C27:0	1	1	2	3	2	2	1.1	NS	NS	NS
CLA t10,c12	2	1	1	2	1	1	0.4	NS	L *	NS
CLA (C18:2cis9,trans11)	31	37	61	31	43	73	13.5	NS	L ***	NS
CLA (Total isomers)	39	45	72	38	52	86	15.8	NS	L ***	NS
Others	75	61	74	59	104	86	23.2	NS	NS	NS
SFA	1431	973	1289	1020	995	1398	275.3	NS	Q+	NS
MUFA	1572	1092	1504	1102	1146	1658	339.0	NS	Q+	NS
PUFA	204	200	242	176	203	264	29.6	NS	L **	NS
n-3 PUFA	74	66	69	62	64	73	8.0	NS	NS	NS
n-6 PUFA	79	81	93	67	77	95	7.5	NS	L ***	NS
PUFA:SFA	0.16	0.21	0.20	0.18	0.22	0.21	0.022	NS	L *, Q *	NS
n-6:n-3 PUFA	1.09	1.26	1.38	1.06	1.22	1.33	0.063	NS	L ***	NS
Desaturase index ^5^	0.49	0.47	0.46	0.47	0.47	0.46	0.013	NS	L+	NS
Atherogenic index ^6^	0.62	0.58	0.58	0.58	0.54	0.56	0.028	NS	L+	NS
Thrombogenic index ^7^	1.27	1.16	1.18	1.22	1.13	1.16	0.052	NS	L * Q *	NS
Total	3285	2333	3120	2363	2457	3419	646.7	NS	Q+	NS

^1^ sed is the standard error of the difference for the W × D interaction with n = 10/group; L, Q are linear and quadratic effects of duration of supplementation, respectively, + = *p* < 0.1, * = *p* < 0.05, ** = *p* < 0.01, *** = *p* < 0.001. ^2^ Co-eluted with C17:0 anteiso. ^3^ Co-eluted with C18:2,10,14 and C19:0. ^4^ Co-eluted with C18:2, 11,14. SFA = saturated fatty acids; MUFA = monounsaturated fatty acids; PUFA = polyunsaturated fatty acids; CLA = conjugated linoleic acid. ^5^ Calculated as (14:1 + 16:1 + 18:1)/14:0 + 16:0 + 18:0 + 14:1 + 16:1 + 18:1). ^6^ Atherogenic index = (12:0 + 4 × 14:0 + 16:0)/(∑MUFA + ∑ n − 6 + ∑n − 3). ^7^ Thrombogenic index = (14:0 + 16:0 + 18:0)/((0.5 × ∑MUFA + 0.5 × ∑n − 6 + 3 × ∑n − 3 + ∑n − 3)/(∑n − 6)).

#### 3.3.2. CLA Isomer Distribution (Table 4)

The predominant CLA isomer was cis9, trans11 representing on average, 80% total CLA. The trans11, cis13 was the next prominent isomer, followed by trans 7, cis9 and trans11, trans13. Of those CLA isomers present at >1% of total CLA, the proportion of trans7, cis9 tended (*p* = 0.09) to be lower while the proportions of trans9, trans11 and trans11, trans13 were higher in LM from cattle offered the US ration during the winter. Increasing the duration of concentrate supplementation linearly increased the proportion of cis9, trans11 and trans7,cis9 and linearly decreased the proportion of trans11, cis13, trans9, trans11, trans11, trans13 and trans12, trans14. There was an interaction between the winter ration and concentrate supplementation (linear) for trans8, cis10 such that the decrease with increase in the duration of concentrate supplementation was greater in LM from animals offered the WO ration during the winter.

**Table 4:** The conjugated linoleic acid (CLA) isomer profile (g/100 g CLA) in *longissimus* muscle of beef heifers.

Winter Ration (W)	Control			Oil-Enriched				Significance ^1^		
Duration of Supplementation (Weeks D)	0	11	22	0	11	22	sed	W	D	W × D
cis-8, cis-10	0.09	0.05	0.03	0.08	0.05	0.03	0.046	NS	NS	NS
cis-9, cis-11	0.04	0.10	0.07	0.21	0.12	0.10	0.093	NS	NS	NS
cis-10, cis-12	0.03	0.02	0.04	0.02	0.05	0.11	0.001	NS	NS	NS
cis-11, cis-13	0.02	0.01	0.01	0.01	0.02	0.01	0.007	NS	NS	NS
cis-12, cis-14	0.01	<0.01	<0.01	<0.01	<0.01	<0.01	0.003	+	L *	*
cis-9, trans-11	77.43	80.30	82.86	77.89	80.55	83.78	1.198	NS	L ***	NS
cis-11, trans-13	0.63	0.48	0.36	0.58	0.47	0.35	0.063	NS	L ***	NS
cis-12, trans-14	0.09	0.09	0.05	0.07	0.07	0.05	0.021	NS	L *	NS
trans-6, cis-8	0.02	0.04	0.02	0.07	0.02	0.03	0.017	NS	NS	*
trans-7, cis-9	3.94	4.56	4.49	4.33	4.60	4.60	0.208	+	*	NS
trans-8, cis-10	2.04	1.96	1.69	2.38	1.98	1.75	0.100	+	L ***	* L
trans-9, cis-11	1.77	1.72	2.31	1.83	1.86	1.53	0.576	NS	NS	NS
trans-10, cis-12	0.13	0.36	0.18	0.17	0.16	0.10	0.064	*	Q *	* Q
trans-11, cis-13	5.06	3.28	2.54	4.56	3.38	2.59	0.342	NS	L *** Q+	NS
trans-12, cis-14	0.60	0.47	0.38	0.55	0.46	0.39	0.050	NS	L ***	NS
trans-13, cis-15	0.01	0.01	<0.01	<0.01	<0.01	<0.01	0.003	NS	NS	NS
trans-7, trans-9	0.15	0.15	0.18	0.17	0.18	0.14	0.022	NS	NS	* L
trans-8, trans-10	0.15	0.22	0.26	0.18	0.23	0.22	0.027	NS	L ***	NS
trans-9, trans-11	1.71	1.65	1.54	1.46	1.45	1.28	0.091	***	L *	NS
trans-10, trans-12	0.32	0.42	0.40	0.36	0.37	0.34	0.048	NS	NS	NS
trans-11, trans-13	3.10	1.89	1.23	2.23	1.64	0.96	0.263	*	L ***	NS
trans-12, trans-14	2.61	2.18	1.34	2.82	2.30	1.62	0.568	NS	L **	NS
trans-13, trans-15	0.06	0.05	0.03	0.05	0.04	0.02	0.016	NS	L *	NS

^1^ sed. is the standard error of the difference for the W × D interaction with n = 10/group; L, Q are linear and quadratic effects of duration of supplementation, respectively, + = *p* < 0.1, * = *p* < 0.05, ** = *p* < 0.01, *** = *p* < 0.001.

#### 3.3.3. Fatty Acid Proportions (Table 5)

Only those fatty acids detected at >0.1 g/100 g fatty acids (for all treatment means) are summarised in Table 5. The proportion of C16:0, C16:0iso, C16:1cis9, C17:0 and C18:1cis11 were higher in LM from cattle offered the US ration during the winter, while the proportions of C18:1trans11, C18:1trans10, C18:1trans12, C18:1trans9, C20:0, CLA cis9, trans11 and total CLA isomers were lower. Increasing the duration of concentrate supplementation linearly increased the proportions of C17:0iso + C16:1trans9 (also quadratic), C18:1trans11, C18:1cis16, C18:1trans10, C18:1trans12, C18:1trans9, C18:1trans16, C18:2 10,13, LA, C18:2trans11,cis15, C20:0, C20:4cis8,cis11,cis14,cis17 (quadratic only), C22:0, DHA (also quadratic),CLA cis9, trans11, total CLA isomers and PUFA (also quadratic).

Increasing the duration of concentrate supplementation linearly decreased the proportions of C15:1, C16:1trans12 (also quadratic), C16:0 (also quadratic), C16:0iso, C16:1cis9 (also quadratic), C16:2cis9, cis12, C17:0, C18:0, C18:1cis9, LNA and SFA. The pattern of response to the duration of concentrate supplementation was quadratic for C20:4cis5,cis8,cis11,cis14 and n-3 PUFA proportions such that they were higher after 11 weeks supplementation but not after 22 weeks of supplementation. 

There was an interaction between the winter ration and concentrate supplementation (linear) for the proportion of C16:1trans7 + trans8 such that it increased with the duration of concentrate supplementation in LM from animals offered the US but deceased with the duration of concentrate supplementation in LM from animals offered the WO ration during the winter. There was an interaction between winter ration and concentrate supplementation (linear) for the proportion of C20:1such that the increase with the duration of concentrate supplementation was greater in LM from animals offered the US ration during the winter.

**Table 5:** The proportion of fatty acids (g/100 g fatty acids) in *longissimus* muscle of beef heifers.

Winter Ration (W)	Control			Oil-Enriched				Significance ^1^		
Duration of Supplementation (Weeks D)	0	11	22	0	11	22	sed	W	D	W × D
C14:0	2.07	1.93	2.06	1.79	1.72	2.00	0.203	NS	NS	NS
C14:1	0.62	0.53	0.57	0.55	0.49	0.52	0.076	NS	NS	NS
C15:0	0.54	0.53	0.50	0.52	0.51	0.52	0.035	NS	NS	NS
C15:0anteiso	0.24	0.23	0.23	0.23	0.22	0.22	0.018	NS	NS	NS
C15:1	0.21	0.19	0.19	0.19	0.17	0.18	0.013	+	L *	NS
C16:1trans12	0.21	0.16	0.16	0.21	0.16	0.15	0.016	NS	L **, Q *	NS
C16:0	24.23	22.91	23.70	23.24	21.30	21.67	0.545	*	L *** Q *	NS
C16:0iso	0.20	0.17	0.16	0.16	0.15	0.15	0.010	**	L **	NS
C16:1cis9 ^2^	3.44	2.80	2.84	2.79	2.55	2.76	0.215	*	L * Q *	NS
C16:1trans7 + trans8	0.41	0.45	0.46	0.46	0.45	0.43	0.029	NS	NS	* L
C16:2cis9,cis12	0.21	0.21	0.12	0.21	0.21	0.16	0.054	NS	L *	NS
C17:0	1.05	0.97	0.95	0.99	0.92	0.89	0.030	*	L ***	NS
C17:0iso + C16:1trans9	0.19	0.34	0.31	0.25	0.38	0.37	0.046	+	L *** Q ***	NS
C17:1cis9	0.51	0.52	0.52	0.51	0.51	0.53	0.045	NS	NS	NS
C18:0	14.50	14.20	13.82	15.40	14.46	14.17	0.662	NS	L *	NS
C18:1trans11	2.57	5.88	7.84	4.65	6.35	8.34	0.628	*	L ***	L+
C18:1cis15 ^3^	0.15	0.16	0.16	0.15	0.18	0.17	0.013	NS	L+,Q+	NS
C18:1cis16	0.29	0.32	0.36	0.30	0.33	0.35	0.026	NS	L **	NS
C18:1cis11	0.85	0.52	0.67	0.47	0.52	0.44	0.143	*	NS	NS
C18:1cis12	0.15	0.14	0.15	0.06	0.16	0.14	0.058	NS	NS	NS
C18:1cis13	0.22	0.17	0.20	0.18	0.18	0.16	0.030	NS	NS	NS
C18:1cis9	34.54	31.05	29.58	32.99	30.17	29.60	1.283	NS	L ***	NS
C18:1trans10	0.24	0.47	0.54	0.39	0.59	0.58	0.083	*	L *** Q+	NS
C18:1trans12	0.20	0.45	0.62	0.39	0.68	0.73	0.083	**	L **	NS
C18:1trans13	2.06	1.42	1.42	1.18	1.63	1.36	0.394	NS	NS	NS
C18:1trans16	0.25	0.30	0.51	0.26	0.28	0.29	0.026	NS	L *	NS
C18:1trans9	0.13	0.22	0.30	0.22	0.26	0.32	0.027	**	L ***	NS
C18:2,10,13 ^4^	0.25	0.29	0.32	0.26	0.31	0.34	0.023	NS	L ***	NS
C18:2cis9,cis12	2.03	2.89	2.78	2.34	2.69	2.65	0.353	NS	L *	NS
C18:2trans11,cis15	0.38	0.51	0.54	0.42	0.49	0.51	0.035	NS	L ***, Q+	NS
C18:3cis9,cis12,cis15	1.07	1.05	0.77	1.12	0.95	0.74	0.124	NS	L ***	NS
C20:0	0.11	0.17	0.22	0.16	0.20	0.25	0.018	**	L ***	NS
C20:1	0.14	0.32	0.40	0.21	0.29	0.37	0.032	NS	L ***	* L
C20:4cis8,cis11,cis14,cis17	0.07	0.13	0.12	0.10	0.12	0.12	0.025	NS	NS	NS
C20:4cis5,cis8,cis11,cis14	0.43	0.59	0.42	0.50	0.60	0.39	0.134	NS	Q *	NS
C20:5cis5,cis8,cis11,cis14,cis17	0.28	0.42	0.32	0.43	0.44	0.34	0.094	NS	NS	NS
C22:1cis11	0.02	0.12	0.22	0.08	0.11	0.20	0.025	NS	L ***	* L
C22:5cis7,cis10,cis13,cis16,cis19	0.42	0.52	0.37	0.54	0.53	0.37	0.102	NS	Q+	NS
C22:6 cis4,cis7,cis10,cis13,cis16,cis19	0.02	0.10	0.09	0.05	0.11	0.10	0.027	NS	L * Q *	NS
CLA (C18:2cis9,trans11)	0.97	1.51	1.88	1.29	1.68	2.14	0.145	*	L ***	NS
CLA (Total isomers)	1.23	1.83	2.24	1.61	2.04	2.50	0.161	*	L ***	NS
Others	2.77	3.07	2.79	2.78	4.75	3.11	0.925	NS	NS	NS
SFA	43.44	41.82	41.29	43.14	40.22	40.60	1.005	NS	L **	NS
MUFA	47.15	46.21	47.58	46.22	46.12	47.74	1.235	NS	NS	NS
PUFA	6.71	8.89	8.35	7.85	8.91	8.55	0.832	NS	L * Q *	NS
n-3 PUFA	2.43	2.96	2.43	2.85	2.88	2.43	0.347	NS	Q *	NS
n-6 PUFA	2.66	3.75	3.41	3.06	3.56	3.28	0.511	NS	Q+	NS

^1^ sed. is the standard error of the difference for the W D interaction with n = 10/group; L, Q are linear and quadratic effects of duration of supplementation, respectively, + = *p* < 0.1, * = *p* < 0.05, ** = *p* < 0.01, *** = *p* < 0.001. ^2^ Co-eluted with C17:0 anteiso; ^3^ Co-eluted with C18:2,10,14 and C19:0. ^4^ Co-eluted with C18:2, 11,14. SFA = saturated fatty acids; MUFA = monounsaturated fatty acids; PUFA = polyunsaturated fatty acids; CLA = conjugated linoleic acid.

### 3.4. Muscle Colour Stability and Sensory Characteristics

The TBARS values (high pH samples excluded) at the start of retail display averaged 0.35 mg malonaldehyde/kg meat and did not differ between treatments. After 10 days of display, the TBARS values were 1.20, 1.52, 1.74, 0.89, 1.32 and 0.96 mg malonaldehyde/kg meat (sed = 0.329) and did not differ between treatments. 

**Figure 1:**
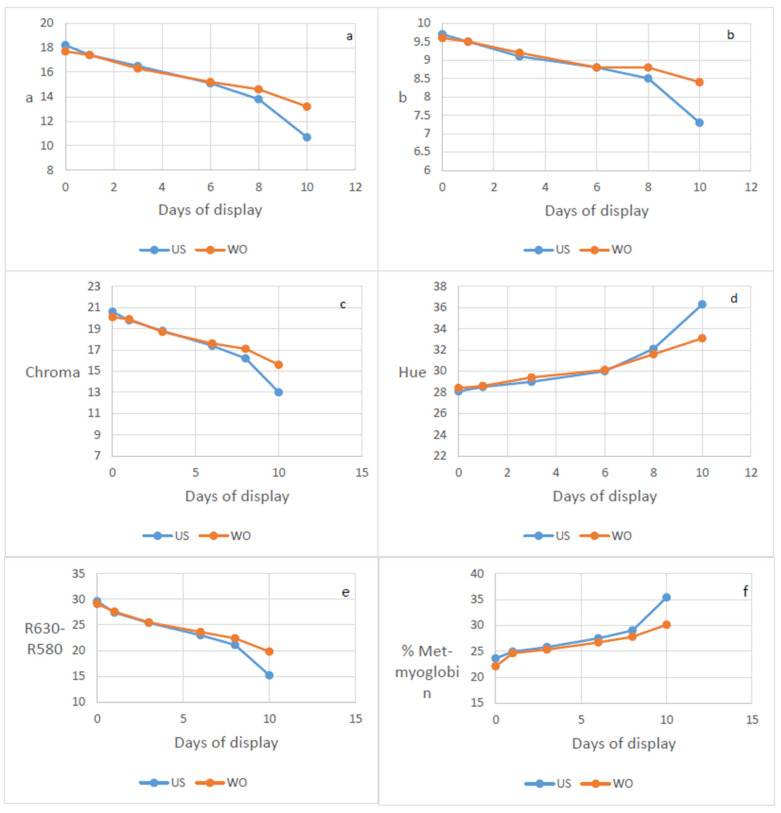
Redness (**a**), yellowness (**b**), chroma (**c**), hue (**d**), reflectance difference (**e**) and % metmyoglobin (**f**) during retail display of longissimus muscle from beef heifers offered either an unwilted grass silage and a barley/soyabean concentrate (US) or wilted grass silage and a concentrate containing sunflower oil and fish oil (WO) ration during the winter prior to finishing at pasture.

Colour variables during retail display are shown in Figure 1. There were no effects of supplementation at pasture and no interactions between the pasture phase and the winter ration for any colour-related variables. The redness (Figure 1a), yellowness (Figure 1b), chroma (Figure 1c) and R_630_-R_580_ (Figure 1e) of LM decreased and hue (Figure 1d) and percentage metmyoglobin (Figure 1f) increased during aerobic display. There was a winter ration by time of display interaction for all variables in Figure 1, which mainly reflected the differences at day 10 of display where LM from animals offered the oil-enhanced winter ration was more colour stable.

Muscle sensory characteristics are shown in Table 6. Muscle from cattle offered the US ration during the winter tended (*p* = 0.06) to be rated more abnormal than muscle from cattle offered the WO ration. Increasing the duration of concentrate supplementation linearly increased the ratings for juiciness, beef (also quadratic), greasy and overall liking and linearly decreased the ratings for fishy and cardboard (quadratic only). There was an interaction between winter ration and concentrate supplementation (quadratic) such that the rating for rancid was lower in LM from cattle offered the concentrate for 11 weeks after receiving the US ration in the winter but was higher in LM from cattle offered the concentrate for 11 weeks after receiving the WO ration in the winter.

### 3.5. Lipid-Related Gene Expression (Table 7)

The expression of SREBP1 was lower and the expression of PPARγ tended (*p* = 0.067) to be lower in LM from cattle offered the US ration during the winter. Increasing the duration of concentrate supplementation linearly increased the expression of the SCAP and tended (quadratic, *p* = 0.098) to decrease the expression of the SREBP1. 

When adjusted for treatment effects, there were few significant correlations between gene expression and the concentration of important fatty acids in LM. Expression of PPARγ tended to be positively correlated with EPA concentration (*p* = 0.065, r = 0.280) and negatively correlated with the desaturase (*p* = 0.078, r = −0.269) ratio. Expression of SCAP was negatively correlated with the EPA (r = −0.359), C20:4cis5,cis8,cis11,cis14 (r = −0.397) and C20:4cis8,cis11,cis14,cis17 (r = −0.360) concentrations and with the PUFA:SFA (r = −0.350) and omega-6:omega-3 PUFA (*p* = 0.074, r = −0.281) ratios. Expression of SCAP was positively correlated with C18:2trans12,cis15 (*p* = 0.052, r = 0.306) and the atherogenic index (r = 0.313). SCD was negatively correlated with C16:2cis9,cis12 (r = −0.313), C18:2cis9,cis12 (r = −0.327), C22:5 (r = −0.415) and omega-6 PUFA (r = −0.343) concentrations. SREBP was positively correlated with the C20:2cis1,cis14 concentration (r = 0.318).

**Table 7:** Gene expression ^1,2^ in *longissimus* muscle of beef heifers.

Winter Ration (W)		Control			Oil-Enriched				Significance ^4^		
Duration of Supplementation (Weeks D)	λ ^3^	0	11	22	0	11	22	sed	W	D	W × D
FADS2	0.25	0.95	0.97	0.99	0.93	1.19	1.12	0.194	NS	NS	NS
FAS	In	1.10	0.91	1.02	0.74	1.02	1.28	0.291	NS	NS	NS
LPL	In	0.87	0.91	1.42	0.89	0.92	1.09	0.335	NS	NS	NS
PPARα	In	1.04	1.11	0.95	1.02	0.78	0.99	0.302	NS	NS	NS
PPARγ	ln	1.05	1.42	0.24	2.10	1.99	1.46	1.099	+	NS	NS
SCAP	ln	0.66	0.90	2.01	0.26	2.86	6.82	1.315	NS	L *	NS
SCD	In	0.43	0.41	1.34	1.07	3.13	0.70	0.858	NS	NS	+
SREBP1	In	0.72	0.21	0.53	3.32	0.96	2.86	1.104	*	Q+	NS

^1^*FADS2 =* Fatty acid desaturase 2/delta-6 fatty acid desaturase; *FAS =* Fatty acid synthase; *LPL* = lipoprotein lipase; *PPARα* = Peroxisome proliferator activated receptor alpha; *PPARγ =* Peroxisome proliferator-activated receptor gamma; *SCAP =* SREBP chaperone; *SCD* = Stearoyl-CoA desaturase/delta-9-desaturase; *SREBP1 =* Sterol regulatory element binding transcription factor 1. ^2^ Values are back-transformed means with the sed of the lambda transformed data. ^3^ Lambda transformation^. 4^ sed is the standard error of the difference for the W × D interaction with n = 10/group; L, Q are linear and quadratic effects of duration of supplementation, respectively, + = *p* < 0.1, * = *p* < 0.05.

### 3.6. Fatty Acid Composition of LM Lipid Fractions

#### 3.6.1. Neutral Lipid Fraction (Table 8)

Only those fatty acids detected at >0.1/100 g fatty acids (for all treatment means) are summarised in Table 8. The proportion of C15:0iso, C16:0, C16:0iso, C16:1cis9, C17:0, LA and LNA and the omega-6:omega-3 PUFA ratio were higher in LM from cattle offered the US ration during the winter, while the proportions of C18:1trans9, C18:1trans12, C18:1trans10, C20:0, CLA cis9, trans11 and total CLA isomers and the PUFA:SFA ratio were lower.

There tended (*p* = 0.067) to be a quadratic response in the total fatty acid concentration such that the concentration was lower after 11 weeks of concentrate supplementation (2081 mg/100 tissue) compared to none (2533 mg/100 g tissue) or 22 weeks (2997 mg/100 g tissue). Increasing the duration of concentrate supplementation linearly increased the proportions of C17:0iso + C16:1trans9, C18:1cis15, C18:1cis16, C18:1trans12, C18:1trans9, C18:1trans16, C18:2 10,13, LA, C18:2trans11,cis15, C20:0, C18:1trans10 (also quadratic), C20:1 (also quadratic), CLA cis9, trans11, total CLA isomers, MUFA, omega-6 PUFA and PUFA and the PUFA:SFA, omega-6:omega-3 PUFA and LA:LNA (also quadratic) ratios. 

Increasing the duration of concentrate supplementation linearly decreased the proportions of C15:0iso, C16:1trans12, C16:0 (also quadratic), C16:0iso, C16:1cis9, C17:0, C18:0, C18:1cis9, LNA and SFA. The long-chain PUFA, EPA and DHA were below the limit of detection.

There was an interaction between the winter ration and concentrate supplementation (linear) for the proportions of C18:1trans11, C20:1 and C22:1cis11 such that the increase with the duration of concentrate supplementation was greater in LM from animals offered the US ration during the winter. 

**Table 8:** Proportions of fatty acids in the neutral lipid fraction of *longissimus* muscle of beef heifers.

Winter Ration (W)	Control			Oil-Enriched				Significance ^1^		
Duration of Supplementation (Weeks D)	0	11	22	0	11	22	sed	W	D	W × D
C14:0	2.25	2.19	2.27	2.03	1.97	2.23	0.195	NS	NS	NS
C15:0	0.59	0.58	0.54	0.58	0.56	0.56	0.040	NS	NS	NS
C15:0anteiso	0.26	0.25	0.24	0.25	0.25	0.24	0.020	NS	NS	NS
C16:0	24.31	23.08	22.63	23.10	21.28	21.75	0.563	**	L * Q *	NS
C16:0iso	0.21	0.19	0.18	0.18	0.17	0.17	0.010	*	L ***	NS
C16:1cis9 ^2^	3.65	3.07	3.06	3.00	2.81	2.96	0.218	*	L *	NS
C17:0 + C16:1trans9	1.12	1.05	1.00	1.07	1.00	0.95	0.031	*	L ***	NS
C17:0iso	0.13	0.16	0.19	0.17	0.18	0.22	0.029	NS	L *	NS
C18:0	15.58	15.59	14.62	16.92	15.93	15.17	0.874	NS	L *	NS
C18:1trans11	2.80	6.55	8.47	5.20	7.07	9.10	0.635	*	L ***	L *
C18:1cis15 ^3^	0.16	0.19	0.18	0.17	0.21	0.18	0.014	NS	Q *	NS
C18:1cis16	0.30	0.34	0.38	0.33	0.35	0.38	0.027	NS	L **	NS
C18:1cis11	0.86	0.44	0.59	0.43	0.47	0.39	0.157	NS	NS	NS
C18:1cis13	0.24	0.20	0.22	0.21	0.20	0.18	0.031	NS	NS	NS
C18:1cis9	35.06	32.21	30.78	33.73	31.34	30.51	1.284	NS	L ***	NS
C18:1trans12	0.23	0.50	0.68	0.45	0.78	0.81	0.087	***	L ***	NS
C18:1trans13	2.39	1.62	1.55	1.34	1.82	1.49	0.461	NS	NS	NS
C18:1trans16	0.27	0.33	0.34	0.29	0.33	0.32	0.029	NS	L *	NS
C18:1trans9	0.15	0.26	0.33	0.25	0.30	0.36	0.030	**	L ***	NS
C18:2 10,13 ^4^	0.29	0.34	0.36	0.29	0.37	0.38	0.019	NS	L *** Q *	NS
C18:2 cis9,cis12 (LA)	1.11	1.23	1.24	1.06	1.07	1.14	0.071	*	L *	NS
C18:2 trans11, cis15	0.41	0.51	0.54	0.46	0.49	0.54	0.030	NS	L ***	NS
C18:3cis9,cis12,cis15 (LNA)	0.70	0.66	0.49	0.65	0.54	0.46	0.044	*	L ***	NS
C20:0	0.13	0.19	0.24	0.18	0.23	0.27	0.018	*	L ***	NS
C14:1	0.70	0.62	0.63	0.63	0.57	0.59	0.074	NS	NS	NS
C16:1trans12	0.22	0.17	0.16	0.22	0.18	0.16	0.016	NS	L ***	NS
C16:1trans7 + trans8	0.40	0.44	0.44	0.45	0.43	0.42	0.031	NS	NS	NS
C16:1cis13	0.12	0.10	0.09	0.10	0.09	0.08	0.129	NS	L *	NS
C17:1cis9	0.57	0.56	0.53	0.57	0.54	0.50	0.049	NS	NS	NS
C18:1trans10	0.28	0.55	0.59	0.45	0.69	0.65	0.090	*	L *** Q *	NS
C20:1	0.15	0.36	0.44	0.24	0.33	0.40	0.033	NS	L *** Q *	L *
C22:1cis11	0.02	0.14	0.24	0.09	0.13	0.23	0.027	NS	L ***	L *
C22:5cis7,cis10,cis13,cis16,cis19	0.12	0.13	0.13	0.13	0.10	0.10	0.021	NS	NS	NS
CLA (C18:2cis9,trans11)	1.05	1.64	2.02	1.43	1.85	2.31	0.143	**	L ***	NS
CLA (Total isomers)	1.34	2.02	2.43	1.81	2.28	2.74	0.161	*	L ***	NS
C15:0iso	0.24	0.23	0.21	0.22	0.20	0.20	0.011	*	L **	NS
Others	2.06	2.32	2.40	2.17	4.06	2.48	1.006	NS	NS	NS
Total mg/100 g muscle)	2968	2012	2869	2097	2150	3126	663.8	NS	Q+	NS
SFA	44.94	43.73	42.28	44.96	41.99	41.96	1.106	NS	L **	NS
MUFA	48.75	48.89	50.00	48.33	48.93	50.03	0.988	NS	L *	NS
PUFA	4.24	5.05	5.33	4.53	5.02	5.52	0.225	NS	L ***	NS
PUFA:SFA	0.09	0.12	0.13	0.10	0.12	0.13	0.007	+	L ***	NS
n-3PUFA	1.44	1.53	1.43	1.44	1.37	1.38	0.073	NS	NS	Q+
n-6PUFA	1.21	1.33	1.33	1.15	1.15	1.23	0.075	+	L *	NS
n-6:n-3 PUFA	0.85	0.87	0.94	0.80	0.85	0.89	0.038	NS	L **	NS
LA:LNA	1.60	1.89	2.53	1.66	1.99	2.50	0.108	NS	L *** Q *	NS

^1^ sed is the standard error of the difference for the W × D interaction with n = 10/group; L, Q are linear and quadratic effects of duration of supplementation, respectively, + = *p* < 0.1, * = *p* < 0.05, ** = *p* < 0.01, *** = *p* < 0.001. ^2^ Co-eluted with C17:0 anteiso; ^3^ Co-eluted with C18:2,10,14 and C19:0; ^4^ Co-eluted with C18:2, 11,14. SFA = saturated fatty acids; MUFA = monounsaturated fatty acids; PUFA = polyunsaturated fatty acids; CLA = conjugated linoleic acid.

#### 3.6.2. Polar Lipid Fraction (Table 9)

Only those fatty acids detected at >0.1 g/100 g fatty acids (for all treatment means) are summarised in Table 9. The proportion of C18:1 was higher in LM from cattle offered the US ration during the winter, while the proportion of C17:1cis7 was lower. Increasing the duration of concentrate supplementation linearly increased the proportions of C17:0iso + C16:1trans9 (also quadratic), C18:1trans11, C18:1cis12 (also quadratic), C18:1trans13, C18:2trans11,cis15, DHA (also quadratic), C17:1cis9,CLA cis9, trans11, total CLA isomers (also quadratic), C20:4cis8,cis11,cis14,cis17, omega-6 PUFA and PUFA and the PUFA:SFA, omega-6:omega-3 PUFA and LA:LNA ratios. 

Increasing the duration of concentrate supplementation linearly decreased the proportions of C16:0 (also quadratic), C16:1cis9, C16:2cis9,cis12, C18:1cis9, LNA C18:3cis9,trans11,cis15 (also quadratic), C22:5 and MUFA.

There was an interaction between the winter ration and concentrate supplementation (linear) for the proportion of C18:1cis 11. Thus, while the proportion of C18:1cis11 was higher in LM from cattle offered the US rations during the winter, the increase with the duration of concentrate supplementation was greater in LM from those animals. For LA, the increase with the duration of concentrate supplementation was greater in LM from cattle offered the US ration during the winter. For C16:1trans 7 + trans8, increasing the duration of concentrate supplementation linearly increased the proportion in LM from cattle offered the US ration during the winter, but linearly decreased the proportion in LM from cattle offered the WO ration during the winter.

**Table 9:** Proportions of fatty acids in the polar lipid fraction of *longissimus* muscle of beef heifers.

Winter Ration (W)	Control			Oil-Enriched				Significance ^1^		
Duration of Supplementation (Weeks D)	0	11	22	0	11	22	sed	W	D	W × D
C14:0	0.32	0.23	0.10	0.07	0.12	0.10	0.136	NS	NS	NS
C15:0	0.14	0.15	0.09	0.11	0.14	0.13	0.053	NS	NS	NS
C16:0	24.19	21.66	23.51	24.73	22.00	23.07	1.162	NS	L * Q *	NS
C16:1cis9 ^2^	1.53	1.09	0.85	1.26	1.06	0.97	0.166	NS	L ***	NS
C16:2cic9,cis12	1.54	1.39	1.32	1.63	1.42	1.46	0.158	NS	L *	NS
C17:0	0.45	0.50	0.44	0.42	0.43	0.37	0.173	NS	NS	NS
C17:0iso+C16:1trans9	0.61	1.43	1.45	0.79	1.60	1.66	0.156	NS	L *** Q ***	NS
C18:0	6.04	6.28	6.00	5.21	6.16	6.50	0.743	NS	NS	NS
C18:1trans11	0.59	1.85	1.63	0.69	1.80	2.15	0.240	NS	L *** Q ***	NS
C18:1cis11	0.80	1.09	1.26	0.76	0.77	0.84	0.118	*	L **	L *
C18:1cis12	0.00	0.43	0.50	0.04	0.28	0.32	0.093	*	L *** Q *	NS
C18:1cis9	29.37	23.46	19.59	27.77	23.99	22.22	1.874	NS	L ***	NS
C18:1trans13	0.09	0.34	0.29	0.23	0.37	0.39	0.063	NS	L * Q *	NS
C18:2cis9,cis12 (LA)	10.11	13.41	17.23	11.16	12.45	15.05	1.056	NS	L ***	L *
C18:2trans1,cis15	0.05	0.58	0.57	0.14	0.49	0.29	0.133	NS	L ** Q **	NS
C18:3cis9,cis12,cis15 (LNA)	4.34	3.40	3.32	4.36	3.36	3.00	0.335	NS	L ***	NS
C20:5 cis5,cis8,cis11,cis14,cis17	2.62	3.07	3.18	3.23	2.86	3.04	0.350	NS	NS	NS
C22:6 cis4,cis7,cis10,cis13,cis16,cis19	0.15	0.74	0.75	0.34	0.73	0.77	0.143	NS	L *** Q *	NS
C16:1trans12	0.10	0.11	0.13	0.12	0.08	0.08	0.051	NS	NS	NS
C16:1trans7+trans8	0.44	0.54	0.61	0.53	0.52	0.39	0.068	NS	NS	L **
C17:1cis9	0.04	0.28	0.35	0.13	0.29	0.59	0.095	*	L ***	NS
C18:3cis9,trans11,cis15	0.15	0.22	0.04	0.15	0.19	0.05	0.052	NS	L ** Q ***	NS
C20:4 cis5,cis8,cis11,cis14	3.95	4.12	3.94	3.51	3.79	3.32	0.448	+	NS	NS
C22:5 cis7,cis10,cis13,cis16,cis19	2.94	2.87	2.60	3.21	2.96	2.56	0.244	NS	L **	NS
CLA (C18:2cis9,trans11)	0.39	0.62	0.60	0.32	0.63	0.71	0.072	NS	L *** Q *	NS
CLA (Total isomers)	0.49	0.70	0.72	0.40	0.75	0.84	0.090	NS	L *** Q *	NS
Others	6.81	6.87	6.23	6.83	8.66	7.31	0.736	*	Q *	NS
Total (mg/100g muscle)	317	321	251	265	307	293	49.7	NS	NS	NS
C20:4 cis8,cis11,cis14,cis17	0.70	0.96	1.20	0.79	0.82	1.10	0.135	NS	L ***	NS
SFA	31.81	30.56	31.74	31.40	30.54	30.96	1.341	NS	NS	NS
MUFA	33.17	29.61	25.49	31.61	29.48	28.31	1.815	NS	L ***	NS
PUFA	28.20	32.78	36.33	30.11	31.17	32.98	2.405	NS	L ***	NS
P:S	0.91	1.10	1.17	0.97	1.03	1.08	0.105	NS	L *	NS
n-3PUFA	11.09	12.06	11.74	12.37	11.64	10.90	0.958	NS	NS	NS
n-6PUFA	15.33	18.94	22.69	15.94	17.65	19.97	1.497	NS	L ***	NS
n-6:n-3	1.38	1.57	1.94	1.30	1.53	1.86	0.064	NS	L ***	NS
LA:LNA	2.34	3.94	5.39	2.66	3.72	5.04	0.338	NS	L ***	NS

^1^ S.e.d. is the standard error of the difference for the W × D interaction with n = 10/group; L, Q are linear and quadratic effects of duration of supplementation, respectively, + = *p* < 0.1, * = *p* < 0.05, ** = *p* < 0.01, *** = *p* < 0.001. ^2^ Co-eluted with C17:0 anteiso. SFA = saturated fatty acids; MUFA = monounsaturated fatty acids; PUFA = polyunsaturated fatty acids; CLA = conjugated linoleic acid.

## 4. Discussion

### 4.1. Context

Beef from grass-based production systems is appreciated by some consumers based in part on the perception of a superior fatty acid profile compared to beef produced in other systems [1,27]. Given the putative health benefits of CLA [4,28] and in particular the cis9, trans11 CLA isomer which is found predominantly in ruminant-derived foods, an increase in the CLA concentration would enhance the value of grass-fed beef further. Since dietary manipulation is the most effective strategy to increase the concentration of CLA in beef [29], all the dietary critical control points must be optimised in an enhanced beef production system. We choose one particular production system, Spring-born suckled heifers slaughtered from pasture in autumn at approximately 20 months of age [30] in which the diet of the animals is predominantly grass-based. The dietary critical control points for increasing the concentration of CLA in beef from these animals are the diet of the mother when the calves are suckling (not considered in this study), the post-weaning indoor diet before turnout to pasture and the pasture finishing phase. Since many of the studies reported to date have focused on enhancing the CLA in the finishing phase of cattle and few have considered a grazing scenario, our ambition was to optimise the latter two critical control points in this production system. The dietary manipulation in the indoor phase was based on Noci et al. [6,7] who demonstrated that sunflower oil inclusion in the concentrate supplement to grass silage–fed steers increased the CLA proportion in muscle and that wilted grass silage increased the concentration of CLA in muscle when compared to unwilted silage, respectively. The dietary manipulation in the pasture finishing phase was based on [8,9] who demonstrated that supplementing grazed grass with sunflower oil alone or with fish oil, respectively, also increased the concentration of CLA. The pathway of biohydrogenation of LA produces CLA directly but biohydrogenation of LA and LNA also includes C18:1trans11 as an intermediate (see [31]). Since most of the CLA found in milk fat resulted from desaturation of C18:1trans11 by the action of Δ9 desaturase [32], the increase in CLA concentration likely reflects an increase in C18:1trans11 accumulation in the rumen and subsequent tissue desaturation. Fish oil has been demonstrated to inhibit the terminal reaction in the biohydrogenation pathway in the rumen and to increase the outflow of C18:1trans11 to the small intestine [33]. In the present study, fish oil was used as a tool to manipulate biohydrogenation rather than to supply longer carbon chain PUFA, although a small proportion of EPA and DHA escaped rumen biohydrogenation and was deposited in tissue.

The feeding strategy was for the cattle to consume similar quantities of energy such that intramuscular fat (IMF) concentration would be similar across treatments and thus avoid confounding treatment effects with fatness [14]. Growth during the indoor phase was close to the target for this system (0.6 kg/day, [30]). Growth at pasture was higher for the supplemented groups reflecting the challenges in implementing the supplementation strategy particularly during a long grazing season. Nevertheless, the differences in the total lipid concentration between treatments only approached significance. It is recognised that if the supplemented grazing groups had unrestricted access to pasture these animals would likely have grown faster which would be closer to a more commercial production system. The implications of this in terms of the fatty acid composition of beef merit examination in a future study.

### 4.2. General Fatty Acid Composition

For individual and classes of fatty acids, presentation of the fatty acid data expressed as a proportion has merit and can allow a more complete comparison with the literature. From a product labelling and ultimately a marketing perspective, concentration data are more relevant for some variables while for others, proportional data are more relevant. Accordingly, the fatty acid data are presented in both forms. Thus, based on the SFA concentration in LM, beef from each production system could be labelled “low in saturated fat” i.e., SFA concentration < 1.5/100 g solid [34]. For MUFA, EU [35] states that “a claim that a food is high in monounsaturated fat may only be made when at least 45% of the fatty acids present” are monounsaturated. Based on the proportion of MUFA in LM, beef from all of the production systems in the present study would meet this claim. For PUFA, EU [34] states that “a claim that a food is high in polyunsaturated fat may only be made when at least 45% of the fatty acids present” are polyunsaturated. Despite the beneficial increase in PUFA observed due to supplementation at pasture, beef from none of the production systems in the present study would meet this claim. 

With regard to ratios of fatty acid classes, there is a recommendation [36] on a desirable ratio of total PUFA: total SFA on a whole diet basis (>0.45), but it does not relate to individual foods. All LM in the present study was below this ratio. From a human nutrition perspective, LM from the supplemented groups has a more desirable PUFA: SFA ratio but the effect is small. Similarly, there is a recommendation [36] on a desirable ratio of total omega-6 PUFA: total omega-3 PUFA on a whole diet basis (<4). While the lower omega-6 PUFA: omega-3 PUFA ratio in LM from the un-supplemented groups may be viewed as positive for “Grass-Fed” beef, the difference is unlikely to be important in this regard.

The concentrations in 100 g of tissue for beef to be labelled as a “source” of omega-3 fatty acids are 300 mg LNA or 40 mg EPA + DHA [37]. In the present study, the highest concentration of LNA was 33 mg/100 g muscle (control indoor diet and no supplementation at pasture) reflecting the higher grass consumption by this group. The highest concentration of EPA + DHA was 12 mg/100 g muscle (oil-rich diet in winter and supplementation for 22 weeks at pasture) reflecting the consumption of fish oil, some of which clearly escaped biohydrogenation in the rumen. While either, on its own, might be viewed as positive for that treatment, and make a contribution to meeting human dietary recommendations, none of the beef in the present study could be labeled a “source” of omega-3 fatty acids as defined by EFSA [37]. 

### 4.3. Conjugated Linoleic Acid

Manipulating the composition of the indoor ration increased the concentration of total CLA isomers, albeit not significantly (52 vs. 58 mg/100 g muscle). When expressed on a proportional basis, thereby adjusting for the difference in the total fatty acid concentration mentioned above, the effect was statistically significant (1.77 vs. 2.05 g/100 g fatty acids) supporting the observations in [6,7]. When compared with the “control” production system, the concentration and proportion of the cis9, trans12 CLA isomer, the isomer most reported in the literature, was 2.4- and 2.2-fold higher in LM from the oil-enriched followed by long-term supplementation at pasture group (86 mg/100 g muscle or 2.5 g/100 g fatty acids). These values are higher than in most previous reports (reviewed by [5,38]). In two studies identified in these reviews where a higher concentration of CLA was reported (156 mg and 134 mg/100 g tissue), the IMF concentration was considerably higher (6.6/100 g and 10.5/100 g tissue, respectively) than in the present study. This reflected the preferential deposition of CLA in the triacylglycerol or NL fraction as demonstrated in the present study (below). As of yet, there is no reference intake for CLA. In a review of the literature, Siurana and Calsamiglia [39] concluded that with respect to human health, “an effective dose would be 0.8 g per day for the anti-carcinogenic effect, 0.6 g per day for the anti-atherosclerotic effect and 3.2 g per day for the reduction of body fat. For other effects, no specific dose has been recommended”. If the contribution to the CLA concentration due to the desaturation of C18:1trans11 to CLAcis9, trans11 in human tissue, (20–25%, [40]) is considered, an average LM steak (200 g) from the oil-enriched followed by long-term supplementation at pasture group could supply approximately 0.3 g CLA. This would make a substantial contribution to the effective dose reported by Siurana and Calsamiglia [39].

While it is recognised that an array of CLA isomers arise from ruminal biohydrogenation of dietary lipids, there are relatively fewer reports on isomers other than cis9, trans11 and trans10, cis12 because they cannot be separated using conventional GC. Because of the potential bioactivity of other CLA isomers [31], and the nature of the supplements, it was considered important to measure their concentration under the dietary scenarios of the present study. Cis9, trans11 was the major isomer and trans11, cis13 and trans7, cis9 the second and third most abundant isomers in the control group in agreement with previous findings for cattle slaughtered from pasture-based production systems [41,42]. The main impact of the dietary modification in the present study was to enrich the LM with the cis9, trans11 isomer (77.4 to 84.8 g/100 g total CLA). While there was some re-ordering of the abundance of the isomers due to the dietary treatments imposed, the concentration of the next abundant isomer was <4.6/100 g total CLA and therefore unlikely to be of relevance to human health. 

### 4.4. Trans Fatty Acids

While the ruminal biohydrogenation pathway was successfully modified to increase the flow of C18:1trans11 from the rumen in the present study, biohydrogenation of dietary PUFA by the rumen microbial system also results in a range of other cis- and trans C18:1isomers. The human health implications of the consumption of trans fatty acids, and in particular their origin, are of current interest [43]. Recent research on ruminally derived trans fatty acids, of which C18:1trans11 predominates (72% of detected C18:1trans isomers in LM from the oil-enriched followed by long-term supplementation at pasture group was C18:1trans11) suggests a positive or neutral effect on human health compared to the detrimental effects of industrially derived trans fatty acids which have a higher proportion of C18:1trans10 and a more diverse profile [44]. However, the duration and daily amount of ruminally derived trans fatty acid consumption required to cause significant effects on human health are still unclear. There is no reference intake value for C18:1trans11 currently. 

Acknowledging the expense and additional management required to supplement cattle for a full grazing season, a shorter period of supplementation was also examined. With regard to the fatty acids of primary interest in this study, CLA and C18:1trans 11, the linear response with duration of supplementation indicates that the full grazing season was necessary to reach the maximum concentrations. The decision on whether a producer might implement this strategy therefore becomes a balance between the added cost of production and the premium achievable in the marketplace.

To explore the site of deposition of fatty acids, the extracted intramuscular lipids were separated into NL and PL fractions. Differences in total intramuscular fatty acid concentrations tend to mainly reflect differences in the size of the NL fraction as the size of the PL fraction is generally quite constant. The preferential incorporation of CLA and C18:1trans10 and C18:1trans11, in particular, into the NL fraction and the preferential incorporation of the longer carbon chain PUFA LA, LNA, EPA and DHA into the PL fraction was also reported by Moreno et al. [14]. This suggests that had a higher target carcass weight, with an associated increase in IMF deposition been chosen, the concentration of CLA in LM would have been even higher. With respect to CLA isomers, the profile was generally similar in the NL and PL fractions, with some evidence that the proportion of the trans11, trans13 and trans12, trans14 isomers are preferentially deposited in the NL fraction (Supplementary Table S2). 

### 4.5. Gene Expression

Using a candidate approach, we examined genes directly involved in lipogenesis, i.e., FAS and LPL or in the regulation of fatty acid metabolism. Dietary lipids act as ligands for a range of receptors which in turn regulate genes coding for transporters and enzymes involved in lipid metabolism [12]. These can result in alterations in the concentration and/or profile of tissue lipids [12]. The ligands for PPARs (key fatty acid metabolic regulators and sensors) encompass a range of exogenous and endogenous lipids, including various fatty acids [45]. The general lack of effect of the production system modifications on the expression of these genes in muscle tissue and their relationships with the concentrations of fatty acids reflects, in part, the minor differences in the latter. Sterol regulatory element-binding proteins (SREBPs), membrane-bound transcription factors that are essential in the regulation of cholesterol, fatty acid and triglyceride biosynthesis and its chaperone SCAP, are essential for promoting nuclear translocation of SREBP1 and activation of FAS gene transcription. The decrease, albeit a quadratic response pattern, in SREBP1 due to supplementation at pasture is consistent with [46]. The opposite patterns for SCAP and SREBP1, an increase and decrease with duration of supplementation at pasture, respectively, indicate an uncoupling between these two regulatory elements. The negative correlations between SCAP and the concentration of EPA and fatty acid indices of relevance to human nutrition are a novel finding and suggest that downregulation of this gene could be a target if the objective was to increase those variables in muscle.

The gene coding for the desaturase enzyme that catalyses the conversion of C18:1trans11 to CLA cis9, trans11 and also C18:0 to C18:1cis 9 (SCD) was of particular interest since part of the dietary strategy sought to increase the supply of C18:1trans11 for subsequent tissue desaturation. While there was a trend towards a decrease in SCD activity based on the desaturase index (calculated from the fatty acid concentrations), due to supplementation at pasture, this was not reflected in SCD gene expression which supports the conclusion that the index is not a good proxy for enzyme activity or indeed gene expression [47]. In contrast, Waters et al. [46] reported a significant decrease in SCD gene expression upon dietary omega-3 PUFA intervention. The expression of the gene coding for the desaturase enzyme that catalyses the first and rate limiting step in the conversion of LA and LNA, FADS2, to highly longer carbon chain unsaturated fatty acids was similarly not affected by the modifications of the production system examined. Overall, the changes in gene expression and therefore their role, if any, in the observed fatty acid profile of muscle were rather modest.

Gene expression was also measured in the subcutaneous lipid as a proxy for the NL fraction for muscle (Supplementary Table S3). As with muscle, there was little effect on SCD expression. While there was an effect on the expression of PPARα, the quadratic response pattern indicates that the timing of sample collection is important in attempting to unravel the relationship between gene expression and the fatty acid profile, i.e., expression tended to be decreased after 11 weeks of supplementation and increased after 22 weeks. As with muscle, SCAP expression was increased by supplementation but only in adipose tissue from animals offered the control ration during the winter which corresponded with an increase in SREBP1 expression. The opposite pattern was observed in adipose tissue for animals offered the oil-rich diet during the winter. These data highlight the challenges in seeking to unravel the role of gene expression in a production system context.

### 4.6. Lipid Oxidation and Colour Stability

Dietary supplementation with PUFA and fish oil in particular can increase lipid oxidation during retail display of beef when compared with beef from un-supplemented cattle [11]. The scale of this effect is influenced by the concentration of long carbon chain PUFA, the concentration of vitamin E (and other antioxidants) and whether display is aerobic or in MAP. The TBARS values for muscle from all groups were below the 2 mg malonaldehyde/kg threshold value for the detection of rancidity in meat by consumers [48]. That the panellists could detect rancidity, albeit at a low level, reflects the training they received prior to asssessment of the beef from the present study. The lack of an effect on lipid oxidation in the present study likely reflects the high vitamin E supply from the oil-rich rations that maintained LM vitamin E concentration, since supplementation with oil-rich feeds without fortification, frequently results in depletion of vitamin E in muscle [49]. Colour stability can be stabilised when muscle has a vitamin E concentration of 3.0–3.5 mg/kg [50] as was the case in this study. CLA has been proposed to have antioxidant properties [51] which may also have contributed to the lipid stability. In support of this, there was relatively little effect on colour stability with the LM from the animals fed the oil-enriched winter ration being more stable after prolonged retail display. Since colour is an important influence on the purchasing decision of the consumer [52], these findings are positive from the perspective of marketing CLA-enhanced beef.

### 4.7. Sensory Characteristics

From a consumer perspective, deleterious effects on the sensory characteristics would diminish the benefits of nutritional enhancement of beef. The higher score for greasiness and beef flavour may be related to the differences in total fatty acid concentrations [53] while the lower score for “fishy” likely reflects the decrease in LNA concentration [48]. That panellists rated LM from the group offered the control winter ration and supplemented for 11 weeks highest for rancidity was unexpected in view of the TBARS values discussed above. Nevertheless, overall, there were relatively minor effects on the measured sensory characteristics of beef which can be viewed as a positive finding as confirmed in the hedonic score for overall liking. It is recognised that while this is an indication of preference by the panel, the assessors cannot be considered typical consumers because of the training they have received in meat assessment and this finding needs confirmation using untrained consumers.

## 5. Conclusions

Modification of the diet of cattle within a grass-based 20-month heifer beef production system resulted in beef that had a CLA concentration that was higher, at a comparable intramuscular fatty acid concentration, than previously reported. When also accounting for the conversion of the enhanced C18:1trans11 concentration, consumption of this beef would make a substantial contribution to the quantity of CLA suggested to have a positive effect on consumer health. That the lipid and colour stability and sensory characteristics of this beef were generally not negatively affected, is a positive result from the perspective of marketing such nutritionally enhanced beef. Since the concentration of both CLA and C18:1trans11 was linearly increased with the duration of supplementation, the decision for the beef producer on the implementation of the strategies explored in this study is based on the added cost of production and the premium achievable in the marketplace. Identification of the latter is an important subject for future research on the topic of CLA-enhanced beef.

## Figures and Tables

**Table 6:** Sensory characteristics of *longissimus* muscle of beef heifers.

Winter Ration (W)	Control			Oil-Enriched				Significance ^1^		
Duration of Supplementation (Weeks D)	0	11	22	0	11	22	sed	W	D	W × D
Attributes ^2^										
Toughness	62.9	58.0	57.9	59.9	62.4	56.8	5.89	NS	NS	NS
Juiciness	38.4	45.3	47.6	39.7	40.7	43.7	3.51	NS	L **	NS
Beef	38.4	37.5	40.8	39.0	38.3	45.7	2.70	NS	L * Q *	NS
Abnormal	23.0	22.7	21.7	18.7	21.7	17.6	2.37	NS	NS	NS
Greasy	12.8	15.0	16.0	13.3	15.4	16.5	1.85	NS	L **	NS
Bloody	12.5	12.5	12.3	12.1	11.4	12.4	1.56	NS	NS	NS
Livery	12.1	11.2	15.4	12.1	12.8	13.1	1.99	NS	NS	NS
Metallic	18.1	15.8	16.6	15.0	15.3	16.5	1.98	NS	NS	NS
Bitter	12.7	12.3	13.6	12.7	13.7	12.1	2.01	NS	NS	NS
Sweet	10.5	11.0	10.7	10.5	12.5	12.6	1.49	NS	NS	NS
Rancid	4.7	4.4	4.9	3.3	6.2	3.4	1.16	NS	Q *	Q *
Fishy	4.2	3.7	3.6	4.0	3.7	3.3	0.48	NS	L *	NS
Acidic	16.6	16.5	15.9	15.2	15.1	15.4	2.51	NS	NS	NS
Cardboard	20.5	15.3	17.2	17.7	17.2	17.5	1.75	NS	Q *	NS
Vegetable/Grass	11.4	10.4	9.5	10.1	12.7	11.6	1.37	NS	NS	NS
Dairy	7.0	7.8	7.9	7.3	7.5	8.2	0.83	NS	NS	NS
Hedonic	29.6	34.6	35.4	31.9	33.5	37.2	3.26	NS	L *	NS
Overall liking										

^1^ sed is the standard error of the difference for the W × D interaction with n = 10/group; L, Q are linear and quadratic effects of duration of supplementation, respectively, * = *p* < 0.05, ** = *p* < 0.01. ^2^ 0 = nil, 100 = extreme, 0–100 mm unstructured intensity line scale.

## Data Availability

The data presented in this study are available on request from the corresponding author.

## Supplementary Materials

The following supporting information can be downloaded at https://www.mdpi.com/article/10.3390/foods11244061/s1: Table S1: Panel of Bovine oligonucleotide primers used for real-time PCR. Table S2: The CLA isomer profile of intramuscular lipid fractions of the longissimus muscle of beef heifers. Table S3: Gene expression in subcutaneous adipose tissue of beef heifers.
