# Supplementary material for: Supplementation with Sunflower/Fish Oil-Containing Concentrates in a Grass-Based Beef Production System: Influence on Fatty Acid Composition, Gene Expression, Lipid and Colour Stability and Sensory Characteristics of Longissimus Muscle
Source: Foods. 2022 Dec 15;11(24):4061. doi: 10.3390/foods11244061 (PMC9778207; doi:10.3390/foods11244061)
Supplement: Supplementary file 1 [file foods-11-04061-s001.zip › foods-2087021-supplementary.pdf]

Table S1. Panel of Bovine oligonucleotide primers used for real-time PCR.

Accession No.	Symbol	Name	Forward<br>Reverse	Product Length
AF229356.1	*PPARα*	Peroxisome proliferator activated receptor alpha	CTGGAGCTGGACGACAGTGA<br>GGAGGCCAGGCCGATCT	73
AB075020.1	*SCD*	Stearoyl-CoA desaturase/ delta-9-desaturase	TGCCCACCACAAGTTTTCAG<br>GCCAACCCACGTGAGAGAAG	80
NM_001113302.1	*SREBP1*	Sterol regulatory element binding transcription factor 1	CACCGAGGCCAAGTTGAATAA<br>GTGCTGAAGGAAGCGGATGT	70
NM_181024.2	*PPARγ*	Peroxisome proliferator-activated receptor gamma	GATCTTGACGGGAAAGACGA<br>GGGGACTGATGTGCTTGAAC	104
NM_001101889.1	*SCAP*	SREBP chaperone	GTG TGCCAGGCTGATCCAT<br>CAGGGTCATCCTCAGCTGAAG	69
NM_001075120.1	*LPL*	Lipoprotein lipase	CAAGTCGCCTTTCTCCTGATG<br>TGACCCCCTGGTGAATGTGT	64
NM_001012669.1	*FAS*	Fatty acid synthase	GAGGACGCTTTCCGTTACATG<br>CGTACCTGAATGACCACTTTGC	62
NM_001083444.1	*FADS2*	Fatty acid desaturase 2/ delta-6 fatty acid desaturase	CGCTGGGAGGAGATTCAGAA<br>TGTAGACCTTGCGATCGATGAC	70
NM_001012682	*RPL0*	Ribosomal protein	TGGTGCTGATGGGCAAGAA<br>GCCGGGTTGTTTTCCAGAT	67
NM_001034034.1	*GAPDH*	Glyceraldehyde-3-phosphate dehydrogenase	GCATCGTGGAGGGACTTATGA<br>GGGCCATCCACAGTCTTCTG	67

Table S2. The CLA isomer profile of intramuscular lipid fractions of the longissimus muscle of beef heifers.

Winter ration (W)	Control			Oil-Enriched				Significance[1]		
Duration of supplementation (weeks D)	0	11	22	0	11	22	sed	W	D	W x D
Neutral lipids										
cis-9, cis-11	0.04	0.11	0.07	0.21	0.17	0.10	0.097	NS	NS	NS
cis-10, cis-12	0.43	0.02	0.04	0.02	0.05	0.11	0.062	NS	NS	NS
cis-9, trans-11	77.27	80.08	82.80	77.84	80.35	83.70	1.246	NS	L***	NS
cis-11, trans-13	0.59	0.44	0.33	0.53	0.42	0.32	0.060	NS	L***	NS
cis-12, trans-14	0.09	0.09	0.05	0.06	0.07	0.05	0.021	NS	L+	NS
trans-7, cis-9	3.95	4.55	4.50	4.32	4.57	4.59	0.209	NS	L*	NS
trans-8, cis-10	2.05	1.98	1.70	2.38	1.98	1.75	0.099	+	L***	L*
trans-9, cis-11	1.79	1.73	2.33	1.83	1.86	1.52	0.594	NS	NS	NS
trans-10, cis-12	0.11	0.33	0.18	0.16	0.15	0.09	0.003	+	Q*	Q*
trans-11, cis-13	5.04	3.27	2.53	4.53	3.39	2.59	0.350	NS	L***	NS
trans-12, cis-14	0.60	0.48	0.38	0.56	0.47	0.40	0.051	NS	L***	NS
trans-7, trans-9	0.15	0.16	0.18	0.17	0.18	0.14	0.022	NS	NS	L*
trans-8, trans-10	0.16	0.22	0.26	0.18	0.23	0.23	0.029	NS	L***	NS
trans-9, trans-11	1.73	1.68	1.56	1.46	1.47	1.30	0.095	***	L*	NS
trans-10, trans-12	0.33	0.44	0.41	0.36	0.39	0.35	0.051	NS	NS	NS
trans-11, trans-13	3.13	1.95	1.24	2.26	1.71	0.98	0.271	**	L***	NS
trans-12, trans-14	2.73	2.33	1.37	2.91	2.48	1.69	0.622	NS	L**	NS
trans-13, trans-15	0.06	0.05	0.03	0.05	0.05	0.02	0.017	NS	L*	NS
polar lipids										
cis-9, cis-11	0.24	0.10	0.12	0.16	0.16	0.11	0.132	NS	NS	NS
cis-11, cis-13	0.24	0.04	0.08	0.08	0.16	0.11	0.097	NS	NS	NS
cis-9, trans-11	79.00	82.82	84.84	79.49	83.04	84.49	1.078	NS	L***	NS
cis-11, trans-13	1.82	1.13	1.48	1.92	1.25	1.12	0.270	NS	L**Q*	NS
cis-12, trans-14	0.12	0.08	0.11	0.11	0.04	0.14	0.053	NS	NS	NS
trans-7, cis-9	3.66	4.57	4.22	4.33	5.19	4.84	0.307	***	L*Q**	NS
trans-8, cis-10	2.22	1.76	1.33	2.24	1.90	1.70	0.219	NS	L***	NS
trans-9, cis-11	1.83	1.72	1.57	1.68	1.85	1.67	0.242	NS	NS	NS
trans-10, cis-12	0.35	0.92	0.33	0.34	0.29	0.29	0.211	+	Q*	Q*
trans-11, cis-13	5.24	3.39	2.97	5.25	3.21	2.93	0.432	NS	L***Q**	NS
trans-12, cis-14	0.41	0.31	0.30	0.33	0.25	0.30	0.076	NS	NS	NS
trans-7, trans-9	0.25	0.20	0.16	0.21	0.20	0.15	0.058	NS	L+	NS

trans-8, trans-10	0.20	0.18	0.24	0.29	0.20	0.15	0.076	NS	NS	NS
trans-9, trans-11	1.45	1.09	1.01	1.29	1.06	0.93	0.123	NS	L***	NS
trans-10, trans-12	0.09	0.18	0.16	0.10	0.19	0.14	0.080	NS	NS	NS
trans-11, trans-13	1.97	0.96	0.65	1.52	0.59	0.48	0.249	NS	L***Q**	NS
trans-12, trans-14	0.71	0.53	0.43	0.67	0.34	0.35	0.154	NS	L**	NS
trans-13, trans-15	0.02	0.01	0.00	0.01	0.01	0.02	0.012	NS	NS	NS

[1] S.e.d. is the standard error of the difference for the W * D interaction with n= 10/group; L, Q are linear and quadratic effects of duration of supplementation, respectively

Table S3. Gene [1,2] expression in subcutaneous adipose tissue of beef heifers

Winter ration (W)		Control			Oil-Enriched				Significance [4]		
Duration of supplementation (weeks D)	λ [3]	0	11	22	0	11	22	sed	W	D	W x D
FADS2	-0.25	0.68	1.02	1.14	0.96	0.72	1.19	0.340	NS	NS	NS
FAS	0.25	1.13	1.14	1.07	0.85	0.98	2.35	0.482	NS	NS	NS
LPL	0.50	1.04	0.93	1.13	1.02	1.17	1.46	0.283	NS	NS	NS
PPARα	0.25	1.14	0.92	1.03	1.04	0.76	1.48	0.222	NS	Q*	NS
PPARγ	0.50	0.85	0.97	1.46	1.26	0.83	1.03	0.231	NS	Q+	NS
SCAP	ln	0.23	0.23	2.91	3.46	4.10	0.47	0.891	*	NS	L*,Q*
SCD	0.50	1.48	0.92	1.51	0.91	0.91	1.90	0.384	NS	Q+	NS
SREBP1	ln	0.26	0.50	1.55	3.94	1.58	0.65	0.942	*	NS	L*

[1] *FADS2* = Fatty acid desaturase 2/delta-6 fatty acid desaturase; *FAS* = Fatty acid synthase; *LPL* = lipoprotein lipase; *PPARα* = Peroxisome proliferator activated receptor alpha; *PPARγ* = Peroxisome proliferator-activated receptor gamma; *SCAP* = SREBP chaperone; *SCD* = Stearoyl-CoA desaturase/delta-9-desaturase; *SREBP1* = Sterol regulatory element binding transcription factor 1.

[2] Values are back-transformed means with the sed of the lambda transformed data.

[3] Lambda transformation

[3] S.e.d. is the standard error of the difference for the W * D interaction with n= 10/group; L, Q are linear and quadratic effects of duration of supplementation, respectively.
